# A Novel Metric to Quantify the Effect of Pathway Enrichment Evaluation With Respect to Biomedical Text-Mined Terms: Development and Feasibility Study

**DOI:** 10.2196/28247

**Published:** 2021-06-18

**Authors:** Xuan Qin, Xinzhi Yao, Jingbo Xia

**Affiliations:** 1 Hubei Key Lab of Agricultural Bioinformatics College of Informatics Huazhong Agricultural University Wuhan China

**Keywords:** pathway enrichment, metric, evaluation, text mining

## Abstract

**Background:**

Natural language processing has long been applied in various applications for biomedical knowledge inference and discovery. Enrichment analysis based on named entity recognition is a classic application for inferring enriched associations in terms of specific biomedical entities such as gene, chemical, and mutation.

**Objective:**

The aim of this study was to investigate the effect of pathway enrichment evaluation with respect to biomedical text-mining results and to develop a novel metric to quantify the effect.

**Methods:**

Four biomedical text mining methods were selected to represent natural language processing methods on drug-related gene mining. Subsequently, a pathway enrichment experiment was performed by using the mined genes, and a series of inverse pathway frequency (IPF) metrics was proposed accordingly to evaluate the effect of pathway enrichment. Thereafter, 7 IPF metrics and traditional *P* value metrics were compared in simulation experiments to test the robustness of the proposed metrics.

**Results:**

IPF metrics were evaluated in a case study of rapamycin-related gene set. By applying the best IPF metrics in a pathway enrichment simulation test, a novel discovery of drug efficacy of rapamycin for breast cancer was replicated from the data chosen prior to the year 2000. Our findings show the effectiveness of the best IPF metric in support of knowledge discovery in new drug use. Further, the mechanism underlying the drug-disease association was visualized by Cytoscape.

**Conclusions:**

The results of this study suggest the effectiveness of the proposed IPF metrics in pathway enrichment evaluation as well as its application in drug use discovery.

## Introduction

The rising health issues worldwide and outbreaks of drug resistance have drawn a great amount of attention to new drug development [1]. However, drug development is expensive and time-consuming, and an average of US $800 million [2] to US $1.8 billion [3] and more than 10 years is invested in the development of 1 drug [4]. Improving the efficiency of drug discovery has long been one of the most important research directions and goals of medical research. As per the data in the 2018 edition of the World Health Organization’s International Classification of Diseases and related health problems, there are 31,055 diseases [5]. Direct drug-disease pairing validation will have 85,214,920 drug-disease treatment validations. This highlights the importance of understanding the mechanisms of disease pathology and the action mechanisms of the existing drugs. According to the data released by the US National Food and Drug Administration in 2018, 35,283 types of drugs and 2744 types of effective ingredients have been approved [6]. Therefore, drug repositioning is recommended as a low-cost drug discovery method based on the clinical use of the drug, by which new indications of the marketed drug are discovered and an old drug is repurposed [4,7]. The linking of drugs to diseases via enriched gene sets is the basis of the drug use strategy under pathway enrichment analysis, which has long been an investigative way to unveil the functional interpretation of known gene sets [8,9]. The enrichment analysis mainly relies on the evaluation of the overexpressed gene set in a specific pathway, thereby leading to functional interpretation [10]. Technically, for a given disease or drug, relevant pathway information is publicly available in pathway databases [11]. For humans, the Kyoto Encyclopedia of Genes and Genomes (KEGG) database [12] contains 38,680 *Homo sapiens* genes, and the abundance of data makes the correlation of disease-related genes or drug-related genes possible. In addition, there are multiple ways to identify a relevant gene set for a given disease [13]. While genome-wide association studies [14] or mRNA analysis [15] is the typical method for drug-related knowledge discovery, biomedical natural language processing is an alternative [16]. However, evaluating pathway enrichment in terms of a chosen gene set exclusively generated by a text mining system is still an unsolved issue [17]. The text mining system extracts the drug-related genes from drug-related literature, and pathway enrichment is then subsequently performed upon the text-mined genes. Although it is believed that text mining takes advantage of the abundant information from text resources [18], the diversity rooted from the various text mining systems leads to diversified results and effects in subsequent pathway enrichment. As representatives of the text mining system, PubTator [19] in a co-occurrence manner and the Turku Event Extraction System (TEES) [20] in a more semantic and syntactic manner play an important role in the biomedical named entity recognition and pathway enrichment.

The framework of this study was as follows. First, we used various biomedical text mining strategies to investigate the drug-related gene sets. Second, we designed novel metrics for pathway enrichment of text-mined genes. Here, 7 novel inverse pathway frequency (IPF) metrics were proposed and they were compared with the traditional *P* values. Finally, we performed a case study to show the effectiveness of the IPF metrics in pathway enrichment as well as the promising application of the text mining pipeline for new drug use discovery.

## Methods

### Collection of Rapamycin-Centric Resources

In this paradigm, a drug-centric text resource was obtained to extract the related genes. We set the drug as rapamycin, also known as sirolimus, as the target drug, which is used for the treatment of renal cell carcinoma and malignant lymphoma. Relevant texts and pathway data were collected targeting rapamycin as follows:

1. Text resources: 31,118 abstracts reporting rapamycin were downloaded from PubMed.
2. Rapamycin-related pathway data set: The drug pathway was retrieved from the comparative toxicogenomic database (CTD) [21], in which the KEGG pathway is enriched significantly among genes that interact with the drug or its downstream entity with a significant *P* value. In total, there are 166 pathways that are related to rapamycin.

### Pathway Enrichment Evaluation in Terms of Text-Mined Genes

**Figure 1:**
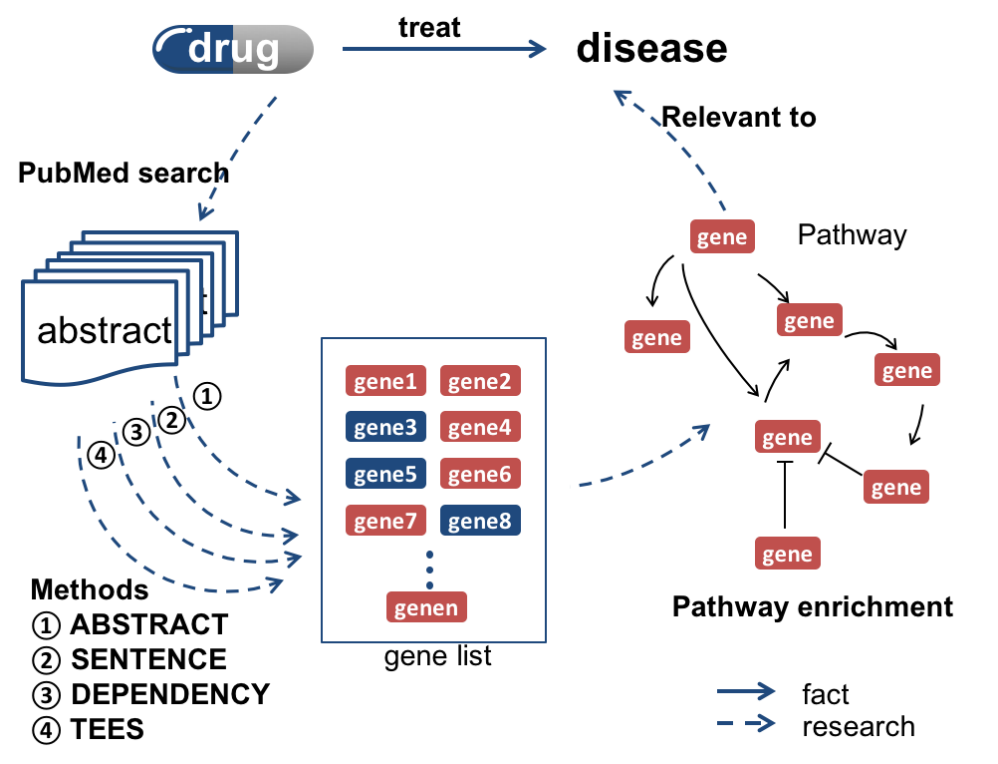
Text mining systems for gene extraction and pathway construction. TEES: Turku Event Extraction System.

As shown in Figure 1, 4 text mining methods were applied to extract the gene pairs in rapamycin-related PubMed texts. They were (1) Method 1: *ABSTRACT* (co-occurrence in abstract) [19], (2) Method 2: *SENTENCE* (co-occurrence in sentence), (3) Method 3: *DEPENDENCY* (under consideration of dependency tree structure) [22], and (4) Method 4: *TEES* (Turku Event Extraction System) [20]. By taking co-occurrence or relation from the above methods, genes were linked to form an undirected pathway. We then proposed 7 types of novel pathway enrichment metrics by introducing various weights to the mined genes. Since the genes were extracted from 4 types of text mining systems, metrics evaluation was compared with respect to different text mining systems. For a given gene set, the candidate pathway is derived from 329 pathways in KEGG. Therefore, the sorted pathways based on *P* values in KEGG enrichment are regarded as the ground truth of pathway enrichment without using the text-mined knowledge. Furthermore, the feasibility of the text mining system for drug mechanism prediction was investigated.

### State-of-the-art Text Mining Methods

**Figure 2:**
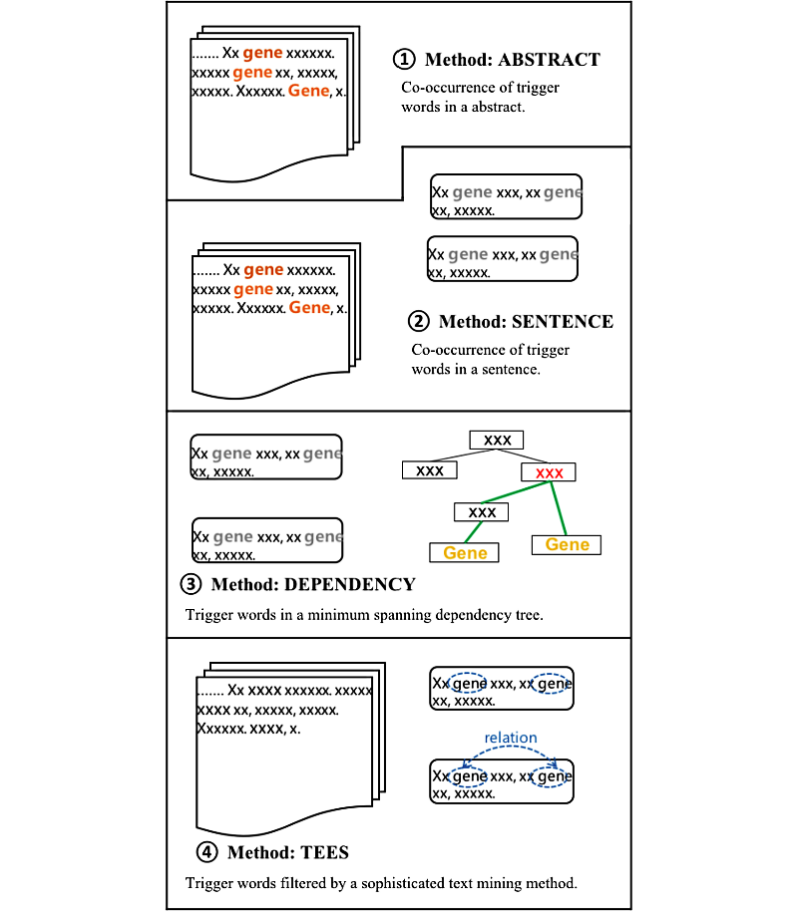
Gene pair extraction rule for the text mining systems.

To extract gene pairs from the abstracts of papers, PubTator [19] and TEES [23] were selected as the 2 baseline text mining tools, which contribute to the following 4 text mining systems (Figure 2):

1. Method 1: *ABSTRACT.* Only abstracts containing the specific drug name were collected. If more than 2 genes showed up in one collected sentence, these genes were extracted and regarded as drug-related genes.
2. Method 2: *SENTENCE.* Similar to the abstract-level extraction rule, gene pairs were extracted based on a sentence co-occurrence rule.
3. Method 3: *DEPENDENCY.* Being stricter than sentence-level gene-pair extraction, the syntactic rule was introduced to restrict the co-occurrence filtering rule. Here, the Stanford parser was used to identity the gene subject or the gene object in a sentence. The gene pair is maintained only when the 2 genes act as sub or obj in the syntactic tree.
4. Method 4: *TEES.* TEES [20] is a sophisticated biomedical relation extraction system, which has been trained over 400,000 linguistics features. TEES is used to extract the genes that have interactions with other genes in drug-related abstracts. Thus, the TEES method provides a set of genes, which shows interaction information in drug-related abstracts.

### Traditional Metrics for Pathway Enrichment

Based on the drug-related abstract text file, 1 text mining tool extracts 1 group of genes. This group of genes is considered to be associated with the drug. For the sake of new drug use discovery, a group of drug-related genes is obtained using a text mining tool. Meanwhile, in the KEGG database, 1 pathway contains a group of genes, which are related to the disease the pathway correlates with. Thus, the matching degree between the drug-related gene group and the disease-related pathway represents the potential of the matching degree between the drug and the disease. ClusterProfile [24] is a known pathway enrichment tool, which applies the *P* value setting for the significance test of the relevant pathway for a given gene set.

Assuming in total that there are *N* background genes related to a specific pathway and there is a given gene set with *k* genes, pathway enrichment is performed to evaluate the significance for the given gene set to be relevant to the specific pathway. The significance value is obtained via chance computation for the given gene set in comparison to a randomly sampled gene set. In random sampling, *k* genes are sampled and *x* out of *k* genes are related to the pathway. Then, the probability for this instance is as follows:



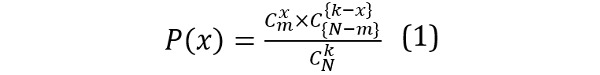



The *P* value used to address the significance of the pathway for the gene set is as follows:



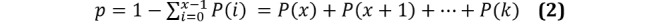



The *P* value as a traditional enrichment metric reflects solid statistical concern in terms of chance computation. It relies on the hypothesis that the chance for each gene belonging to a given gene set is equal. However, this prerequisite is in some cases not met, for example, housekeeping genes have higher chances to appear in any given pathway, while on the contrary, certain specific genes only appear in a specific pathway.

### Proposed Metrics for Pathway Enrichment

#### IPF for a Gene in a Given Pathway

The 4 text mining methods extracted 4 different sets of drug-related genes. Through these gene-drug relations, a bridge between the genes and the drug was established. The aim of this study was to investigate how a drug is associated with its indication through the gene. The next part was to establish the bridge between these genes and the indication. Mature gene-disease relations were easily accessed through KEGG in the form of the KEGG pathway. The KEGG pathway is a collection of manually drawn pathway maps representing the knowledge on the molecular interaction, reactions, and relation network. Thus, a bridge between the genes and the drug was established via KEGG. The whole path in that mechanism was addressed by finding a gene bridge between the drug and its indication. The next step was to evaluate this strategy. We paid attention to which text mining method is more suitable in this strategy. We focused on the drug-related gene set extracted by the text mining method in terms of the quantity and importance. Thus, we needed to define the importance standard of the gene to the indication. The standard of the gene to the indication in this case is based on the KEGG pathway information. One gene specifically shows up in a specific pathway, which means that this pathway can be identified with this gene. In other words, the less pathways a gene appears in, the more important it is to its related pathway. To calculate this situation, we give a value IPF.



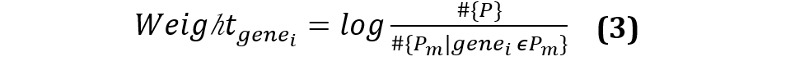



Where *P*={*p*_1_,*p*_2_…..*p*_M_} refers to all KEGG pathways, where *M=#{P}* is the number of pathways in the KEGG database.

{p_m_|gene_i_ ∈ p_m_} refers to a pathway that contains the *i*-th gene, denoted as *gene_i_*. Thus, every gene in the KEGG database receives a basic score. Simply adding all the gene scores together is unfair. Because all pathways show up in KEGG in the form of a map, each map consists of a set of node boxes and severe edges instead of genes and edges. Therefore, we need to figure out how to calculate that score that one text mining method receives from all node boxes in a specific pathway.

#### Enrichment of Text Mining–Based Gene Sets in a Pathway in View of a Gene

Assume *T_t_* is a gene set that contains all of the genes mined by the *t-*th text mining method. In order to evaluate how *T_t_* genes are enriched in a specific pathway, *P_m_*, we define



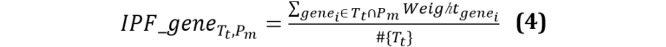



Where *IPF_gene_T_t_,P_m__* considers the number of genes that exist in a pathway as well as the weight of each gene. The sum of the IPFs can be used to evaluate the association of the group of genes to a pathway. By doing this, cumulative associations along with gene weights are represented.

#### Enrichment of Text Mining–Based Gene Sets in a Pathway in View of a Node

In KEGG, a node box in some cases represents 1 set of homologous genes, instead of 1 separate gene. Generally, although there exists more than 1 gene, these genes play the same role. Therefore, even the text mining method digs more than one gene belonging to this pathway but they play the same role in the same node box. We only applied the max gene score to represent the score that this text mining method receives in this node box in this pathway. If *node_j_* is a single node,



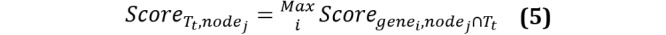



where



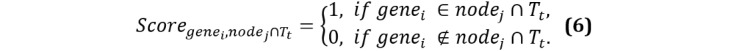



If *node_j_* has E subnodes,



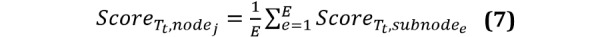



Where *g_i_ ∈ {N_node_j__ ∩ T_t_}, g_i_ = g_max_*

For each *gene_i_*, which belongs to gene set *node_j_* as well as *T_t_*, the maximum IPF*gene_i_* is assigned, which means *gene_i_* belongs to gene set *N_node_j__*.

It is noted that a node box sometimes represents 1 set of protein complex genes that need to work together to play a role in the pathway. Therefore, we applied the sum of all the gene scores that the text mining method received in this node and multiplied it with a coefficient to represent the score that this text mining method receives in this node box in this pathway.



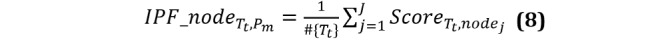



where *∣N_node_j__∣* means the gene number of gene set *N_node_j__*, while ∣*g_i_* ∈ {*node_j_* ∩ *T_t_*}∣ means the gene number of the union of gene set *N_node_j__* and gene set *T_t_*.

#### Enrichment of Text Mining-Based Gene Sets in a Pathway in View of the Shortest Path

Besides the inclusion of genes in 1 node, the graph theory of the node in the pathway should be taken into consideration. In graph theory, the degree of a vertex is the number of edges associated with the vertex. In a pathway graph, one node holding a high degree indicates that this node connects with more vertices. In term of gene, this gene is associated with many genes. Mutations and regulation of the gene affect more genes. In 1 pathway, the more a node shows up in the shortest path between the 2 genes, the more important this gene is in this pathway.

First, assume *SP_node_r_,node_s__* refers to the shortest path between 2 arbitrary nodes, that is, *node_r_* and *node_s_* in pathway *P_m_*, then, we count the occurrence of *node_j_* in *SP_node_r_,node_s__* with respect to *P_m_*.

*Count_node_j_,P_m__* = #{*SP_node_r_,node_s__|node_j_ ∈ SP_node_r_,node_s__*} **(9)**

In addition, *NShortPath_node_r_,node_j_,node_k__* is a binary value, which denotes whether or not *node_j_* appears in the shortest path between *node_r_* and *node_k_*.

Thus, each node in the pathway holds a “count” score. To compare the importance of a node among all the nodes in one pathway, softmax function is applied to *NShortPath_node_r_,node_j_,node_k__*. Here, the softmax function is the gradient logarithmic normalization of the discrete probability distribution of finite terms. The result of softmax is suitable for describing the importance of 1 node in 1 pathway.



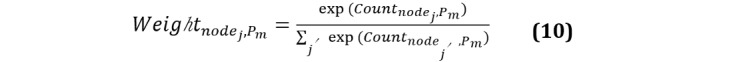



Then, we added all *IPF_node_j__* to represent the total score that the text mining method receives in this pathway,



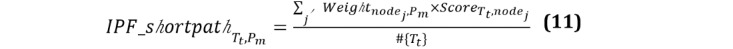



where



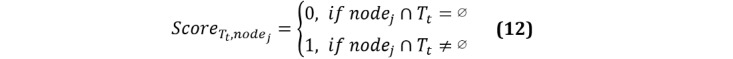



Based on the above discussion on *IPF_gene* (equation 4), *IPF_node* (equation 8), and *IPF_shortpath* (equation 11), we formulate a generalized formula for *IPF_node_T_t_,P_m__*.



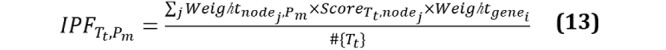



Here, equation (13) summarizes all the above metric considerations and proposes a generalized form of IPF metrics. For instance, *IPF_gene* in equation (4) holds if 1 is assigned to *Weight_node_j_,P_m__*. Equation (12) is assigned to *Score_T_t_,node_j__* Score(*T_t_*, *node_j_*) and equation (3) to *Weight_gene_i__*. The full list of generalized IPF metrics is shown in Table 1.

**Table 1:** The complete inverse pathway frequency metrics list.

Inverse pathway frequency (IPF) metrics	*Weight_node_j_,P_m__*	*Score_T_t_,node_j__*	*Weight_gene_i__*
IPF_gene	1	Equation (12)	Equation (3)
IPF_node	1	Equations (5) and (7)	1
IPF_shortpath	Equation (10)	Equation (12)	1
IPF_shortpath_gene	Equation (10)	Equation (12)	Equation (3)
IPF_shortpath_node	Equation (10)	Equations (5) and (7)	1
IPF_gene_node	1	Equations (5) and (7)	Equation (3)
IPF_gene_node_shortpath	Equations (5), (7), and (10)	Equations (5) and (7)	Equation (3)

## Results

### IPF Metric Comparison Under the Evaluation of Relevance Gene Ranking

**Figure 3:**
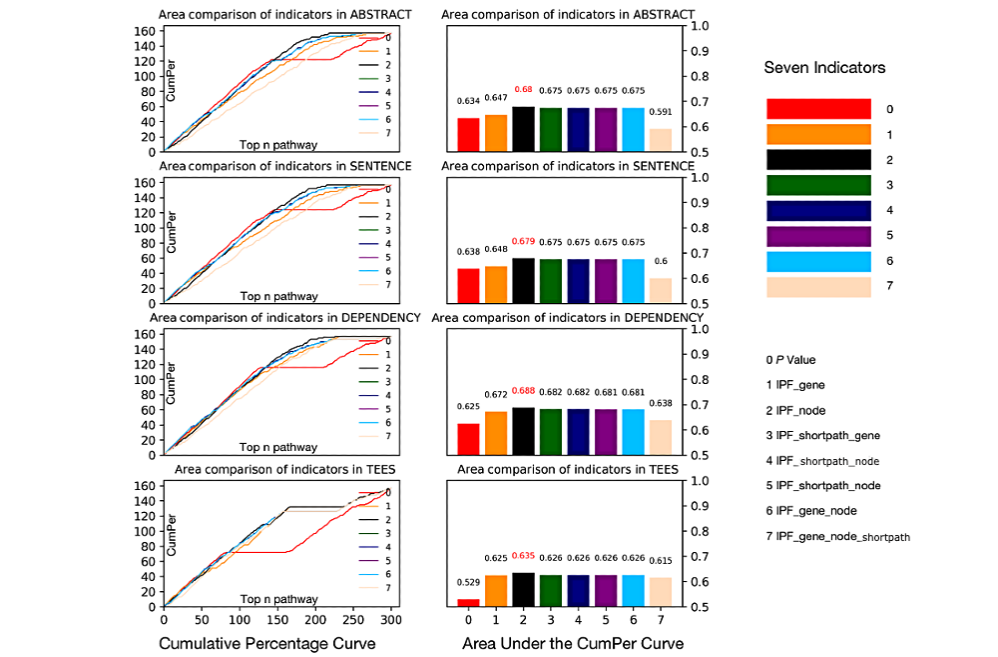
Comparison of the pathway-enrichment metrics based on the rapamycin-related gene set. CumPer: cumulative percentage; IPF: inverse pathway frequency; TEES: Turku Event Extraction System.

We evaluated the effectiveness of IPF metrics by observing the rank counts of topic-related genes obtained from the 4 text mining methods. First, the 4 baseline text mining methods, that is, ABSTRACT, SENTENCE, DEPENDENCY, and TEES, were used to filter the vital genes in rapamycin-related texts. Afterwards, for each gene set obtained by the various text mining methods, 7 IPF metrics and traditional *P* values were used to map to obtain vital pathways and their pathway ranks. We then evaluated the pathway ranks by counting the occurrences of the key CTD pathways depicted in the Methods section. As shown in Figure 3 and Table 2, the x-axis refers to the rank of the enriched pathways and the y-axis refers to the cumulative percentage (CumPer), which is the ratio of the vital CTD pathway among the top i-th enriched pathways.



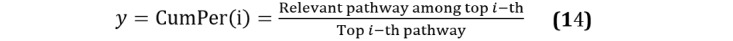



**Table 2:** Comparison of the areas under the cumulative percentage curve for the pathway-enriched methods based on the known rapamycin-related pathway.

Inverse pathway frequency metrics	ABSTRACT	SENTENCE	DEPENDENCY	Turku Event Extraction System
IPF_gene	0.634	0.638	0.628	0.529
IPF_node	0.647	0.648	0.672	0.625
IPF_shortpath	0.680^a^	0.679^a^	0.688^a^	0.635^a^
IPF_shortpath_gene	0.675	0.675	0.682	0.626
IPF_shortpath_node	0.675	0.675	0.682	0.626
IPF_gene_node	0.675	0.675	0.682	0.626
IPF_gene_node_shortpath	0.675	0.675	0.681	0.626
*P* value	.59	.60	.64	.62

^a^Indicates that the area is significantly superior to this text mining method in terms of the pathway enrichment indicator.

The bars from 0 to 8 in the bar plot represent the *P* value and 7 IPF metrics in Table 1, respectively. The results show that genes ranked with *P* values map to less vital pathways than genes from IPF metrics. In detail, the cumulative percentage curves of *P* values are given in the left 4 plots, and it is straightforward to observe that the *y* obtained by the *P* value grades the lowest in all the text mining cases. If computing the area under the cumulative percentage curve, the areas are 0.634, 0.638, 0.625, and 0.529 for *P* values for each case, which are as well the least in all cases. In all, the consistency of the poor performance of the *P* value positively shows the effectiveness of the IPF metric in support of the key pathway enrichment. Furthermore, in all the 7 IPF metrics, the black bar, which represents *IPF_node*, performs the best with the highest value of area under the cumulative percentage curve. It achieves 0.68, 0.679, 0.688, and 0.635 in *ABSTRACT, SENTENCE, DEPENDENCY*, and *TEES*, respectively.

### Artificial Intelligence in Pathway Enrichment

Although the area values among IPF metrics do not differ substantially from each other, the *IPF_node* prevails over the rest of all in a consistent manner. The results show that the *IPF_node* represents the best semantic feature from the view of the natural language processing method and it is the most supportive for vital pathway enrichment.

#### Replication in the Discovery of Efficacy of Rapamycin for Breast Cancer

The discovery of the efficacy of rapamycin was replicated via a pathway enrichment experiment. PubReminer was used to retrieve the research trend of rapamycin and breast cancer drugs. A total of 1502 abstracts were obtained, and the starting time was the year 2000. The experiment was designed to test if the gene interaction of rapamycin could be excavated by the text mining method from literature without reporting the relevance of breast cancer and rapamycin. All the gene pairs in the literature related to rapamycin from the years 1978 to 2000 were excavated, the active genes of rapamycin were obtained, and the enrichment analysis of the strategic gene pathway in this study was carried out. After applying the *IPF_node,* 1640 abstracts of rapamycin prior to the year 2000 were obtained and 243 genes were obtained. Afterwards, a standard pathway enrichment was obtained, and the top 0.5% of the pathways under each enrichment path index was statistically analyzed. As expected, the breast cancer pathway was listed in the enrichment results, and the results indicated that the potential activity of rapamycin can be obtained by enriching the gene pathway by text mining interaction genes.

#### Visualization of the Pharmaceutical Mechanism

The text mining system was investigated to bridge the drug, protein, and disease pathway in order to explore the pharmaceutical mechanism of rapamycin. Starting from the Literature Network application, the disease-related gene network was constructed, and 480 genes obtained by rapamycin-centric text mining were used to highlight the overlapping parts in the breast cancer gene network. All the breast cancer–related genes were collected from the STRING database. According to all the existing databases and text information, each gene was sorted for rapamycin correlation, and in this verification section, 100 breast cancer–related genes from STRING were selected. The breast cancer gene network was constructed according to the gene interaction mentioned in more than 40,000 papers, and the network was constructed using the literature network application program. After gene pathway enrichment analysis, the drug was associated with the pathway and Cytoscape was used for network visualization. In view of the relation between the pathway information and the disease, the drug was further associated with the disease. In order to further analyze the relationship between drugs and diseases, the distribution of the drug-active genes excavated in the disease gene network was analyzed.

**Figure 4:**
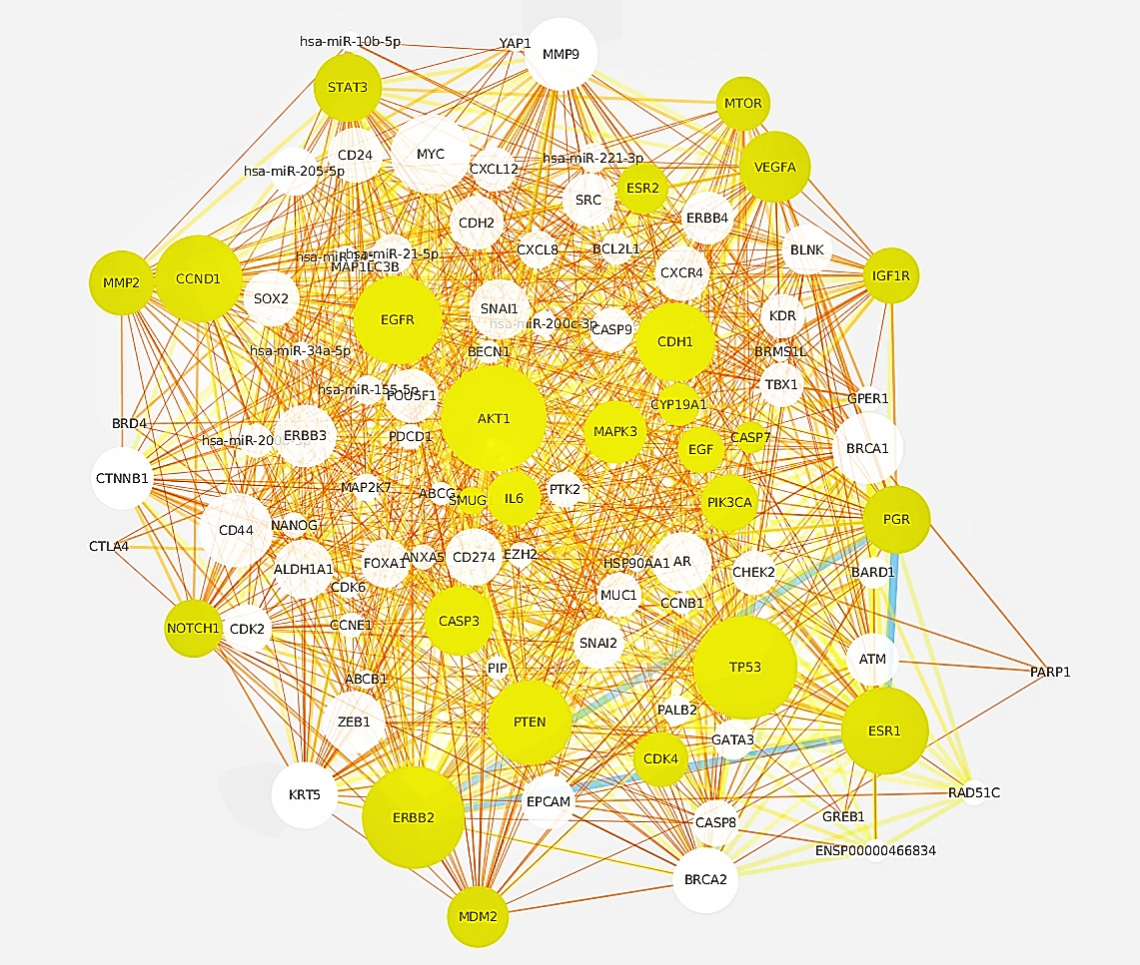
Visualization of the extracted gene pairs from literature.

In order to construct a disease-specific gene network, the genetic relationship of this network in nature was obtained from disease-related abstracts. Since Cytoscape is a high-quality visualization platform for network analysis, a literature network application program based on Cytoscape was applied to address the drug disease associations obtained after pathway enrichment. Figure 4 highlights 38 vital genes plotted as yellow circles, namely, *STAT3, TP53, CDK4, CTLA4, AR, MYC, NOTCH1, IL6, ERBB2, CXCL12, BECN1, IGF1R, CDK2, EGF, ERBB4, MMP9, PIK3CA, CXCL8, ABCB1, EZH2, CDK6, SOX2, AKT1, CDH1, SRC, MTOR, ABCG2, KDR, CCND1, VEGFA, EGFR, ZEB1, ATM, PTEN, CXCR4, ERBB3, MDM2,* and *GATA3*. These 38 genes are based on the intersection of the breast cancer text network and the drug rapamycin-active gene obtained in this strategy. The size of the point in the graph represents the degree of the point, the greater the degree, the larger the point, and the degree in this network is the number of proteins that interact with the protein. The edge thickness in the figure represents the number of sentences that support the protein-protein relationship. The edge color in the figure also represents the number of sentences that support the protein-protein relationship. It can be seen from the figure that the yellow bright spot covers the vast majority of breast cancer gene networks with moderately large spots. The 38 genes were enriched by the *P* value pathway, and 16 of them, that is, *EGFR, IL6, TP53, CDK6, CDK4, PTEN, CDK2, KDR, AKT1, IGF1R, CCND1, VEGFA, PIK3CA, MDM2, MTOR,* and the *MYC* signaling pathway belong to one of the *MTOR* signaling pathways. Among them, *MTOR* is an important gene targeted by rapamycin. The *MTOR* pathway plays an important role in multiple activities of rapamycin and is therefore linked to breast cancer. The reason that literature network is used to construct breast cancer–related network is that the protein interaction involved in constructing the network is obtained from the literature related to breast cancer, and it is the programmed realization of protein interaction based on sentence coexpression in this study. It is convenient for users to quickly construct interactive protein interaction networks based on text relationships. In this study, the breast cancer–related genes obtained from the STRING database were rearranged according to the text information, and the protein interaction information excavated from the text was reflected in the size of the protein gene points. Thus, breast cancer genes were given different weights. It is more convenient to give priority to the location of the active genes under the active conditions defined by the interaction. The overlap of disease and drug-active genes was observed and the possible mechanism of action was speculated.

## Discussion

In this study, all text resources were obtained from a rapamycin-centric literature data set prior to the year 2000, and all predicted drug efficacies for rapamycin were based on knowledge ahead of this timeline. Therefore, it was interesting to “replicate” and evaluate a novel pathway-discovery method in our case study and to investigate the research paradigm based on pathway enrichment. Several studies after the year 2000 provide evidences to show that the mined rapamycin-centric pathway make sense. For example, after Liu et al [25] reported the effect of rapamycin in effectively inhibiting the growth of breast cancer in preclinical and clinical trials, the mechanism of action of rapamycin was elucidated. Rapamycin controls the growth, metabolism, and senescence of cells, as well as cells’ reactions to nutrients, energy levels, and growth factors. *MTOR*, the target of rapamycin, is often upregulated in a variety of cancers, while rapamycin is extremely selective in blocking *MTOR*. Interestingly, our case study pinpointed *MTOR* correctly and made our pathway enrichment method conceivable in the study of breast cancer. Hopefully, the investigation of rapamycin action in the treatment of breast cancer will be propelled by further extensive and abundant text mining results in the future.

In conclusion, this research proposed a group of new pathway enrichment metrics by combining protein-interaction mechanisms, graph theories, information retrieval, and data mining weighting technology and by providing a new idea on pathway enrichment analysis. Moreover, the effectiveness of the best new enrichment metric for rapamycin was analyzed and the new activity of the drug shown by our method is supported by evidence from the literature. This research strategy sheds light on the investigation of the mechanism of action of drugs on diseases by using text-mined genes that are enriched in signaling pathways.

## Abbreviations

CTD: comparative toxicogenomic database
IPF: inverse pathway frequency
KEGG: Kyoto Encyclopedia of Genes and Genomes
TEES: Turku Event Extraction System
